# Available Oocytes rate as a predictor of clinical pregnancy outcomes in controlled ovarian stimulation: A retrospective analysis of 7933 Cycles

**DOI:** 10.12669/pjms.40.11.10094

**Published:** 2024-12

**Authors:** Peiyang Lin, Rongshan Li, Suqin Zhu, Xiuqiong Zheng, Huiling Xu, Beihong Zheng

**Affiliations:** 1 Peiyang Lin Fujian Obstetrics and Gynecology Hospital, Fuzhou, Fujian Province, China; 2 Rongshan Li Reproductive Medicine Center, Fujian Maternity and Child Health Hospital, Affiliated Hospital of Fujian Medical University, 18 Daoshan Road, Fuzhou, Fujian Province 350001, China; 3 Suqin Zhu Reproductive Medicine Center, Fujian Maternity and Child Health Hospital, Affiliated Hospital of Fujian Medical University, 18 Daoshan Road, Fuzhou, Fujian Province 350001, China; 4 Xiuqiong Zheng Reproductive Medicine Center, Fujian Maternity and Child Health Hospital, Affiliated Hospital of Fujian Medical University, 18 Daoshan Road, Fuzhou, Fujian Province 350001, China; 5 Huiling Xu Reproductive Medicine Center, Fujian Maternity and Child Health Hospital, Affiliated Hospital of Fujian Medical University, 18 Daoshan Road, Fuzhou, Fujian Province 350001, China; 6 Beihong Zheng Reproductive Medicine Center, Fujian Maternity and Child Health Hospital, Affiliated Hospital of Fujian Medical University, 18 Daoshan Road, Fuzhou, Fujian Province 350001, China

**Keywords:** Available oocytes rate, Human menopausal gonadotropin, Ovulation stimulation, Ovarian response, Predictive biomarker, Assisted reproductive technology

## Abstract

**Objective:**

Controlled ovarian stimulation (COS) and embryo culture may affect the development and maturation of oocytes obtained by assisted reproduction technologies (ART). This study used the concept of available oocytes rate (AOR) to evaluate the effect of COS and to identify factors that influence oocyte development and clinical pregnancy (CP).

**Methods:**

Medical data of 7933 patients who underwent oocyte retrieval and ART treatments was retrospectively reviewed at Fujian Provincial Maternity and Children’s Hospital from January 2013 to December 2019. Baseline characteristics of patients, concentrations of hormones, as well as endometrial thickness, number of aspirated follicles, retrieved and available oocytes, and CP rates, were analyzed.

**Results:**

Univariate and multivariate analyses showed that basal estradiol (E2) (OR=0.98, p=0.04), endometrial thickness on human chorionic gonadotropin (hCG)-day (OR=1.25, p<0.01), number of follicles aspirated (OR=1.58, p<0.01), oocytes retrieved (OR=0.80, p=0.04), available oocytes (OR=0.48, p=0.04) and AOR (OR=1.18, p<0.01) contributed to CP. The receiver operating characteristic (ROC) curve for the CP rates showed a possible cutoff for the AOR (area under the curve (AUC)=0.788, cut off=34.13%). All 103 cycles with an AOR less than 34% were then selected as Group-A, and 103 cycles were selected as Group-C by 1:1 case-control matching. The human menopausal gonadotropin (HMG) dose was significantly different between Group-A and Group-C (1064.00±1042.01 U vs. 675.00±691.67 U, respectively, p=0.006). The duration of HMG usage was 7.88±4.73 days in Group-A and 5.79±3.59 days in Group-C (p=0.014).

**Conclusions:**

The AOR is an important indicator of oocyte function and is correlated with clinical pregnancy outcomes of fresh cycles. The AOR could objectively predict HMG as a clinically related factor that affected the number and maturation of oocytes for insemination.

**Abbreviations:**
COS: controlled ovarian stimulation;
ART: assisted reproductive technology;
CP: clinical pregnancy;
AOR: available ocytes rate;
E2: estradiol;
FSH: follicle-stimulating hormone;
LH: luteinizing homone;
AUC: the area under the curve;
HMG: human menopausal gonadotropin;
IVF: in vitro fertilization;
ICSI: intracytoplasmic sperm injection;
ET: embryo transfer;
IVM: in vitro maturation;
FET: frozen-thawd embryo transfer;
AFC: antral follicl count;
hCG: human chorionic gonadotrophin;
ROC: receiver oprating characteristic;
Gn: gonadotropi;
AMH: anti-mullerian hormone;
POR: poor ovarian response;
SOR: suboptimal ovarian response;
rLH: recombinant luteinizing hormone;
rFSH: recombinant follicle-stimulating hormone.

## INTRODUCTION

Infertility, defined as an inability to establish a clinical pregnancy (CP) after 12 months of regular, unprotected sexual intercourse, is a global health concern that affects 10-15% of couples of childbearing age worldwide.^1^-^3^ The incidence of female infertility increases with the age of women at conception, economic development, changes in social roles, and increasing psychological pressure.^4^ In vitro fertilization and intracytoplasmic sperm injection and embryo transfer (IVF/ICSI-ET) are common and effective assisted reproductive technologies (ART),^5^,^6^ but their success rate is affected by multiple factors, such as the baseline characteristics of the patients, the quality of gametes, the response to ovarian hyperstimulation, and embryo culture technology (e.g., embryo culture in a time-lapse system or a traditional embryo culture).^7^

Studies show that oocyte quality is crucial for ensuring the success of ART treatment.^8^ However, despite constant optimization of ovulation induction protocols and timing of the HCG triggering and oocyte retrieval, a small percentage of patients still present with a low number of oocytes available for insemination.^9^ Therefore, evaluating the clinical ovulation stimulation effect before fertilization and increasing the number and quality of oocytes available for subsequent insemination is crucial for improving the success rates of ART.

Hormonal markers during the process of controlled ovarian stimulation (COS) are the main indicators for evaluating the effect of ovulation induction,^10^ and the number of oocytes obtained after one ovulation is often used as the measure of ovarian response.^11^,^12^ Patients defined as having a “poor prognosis” can be considered women with expected poor ovarian response to ovarian stimulation in terms of the number of oocytes retrieved. However, this method relies only on the number of retrieved oocytes and does not assess the development of follicles in the process of ovulation induction and the number of oocytes that can be used for insemination. In this study, we have introduced a new concept of the available oocytes rate (AOR). AOR refers to the number of oocytes used for insemination out of the total number of aspirated follicles and, therefore, comprehensively considers the development and maturity of follicles and oocytes.^13^ Our study aimed to explore clinically related factors that may potentially affect the maturation of oocytes. Our results may contribute to developing more suitable ovulation induction programs and reasonable medication guidance for patients with adverse fertility outcomes during COS and improve pregnancy outcomes for these patients.

## METHODS

Medical records of 12599 patients who underwent oocyte retrieval and IVF-ET or ICSI-ET at the Reproductive Medicine Center, Fujian Provincial Maternity and Children’s Hospital, between January 2013 and December 2019 were retrospectively selected.

### Ethics approval:

The human subjects ethics committee of Fujian Maternity and Child Health Hospital approved the study (No. 2023KY161), Date: November 14^th^, 2023.

### Inclusion Criteria:

- Fresh IVF-ET or ICSI-ET cycles
- Complete medical records.

### Exclusion Criteria:

- Patients who underwent frozen-thawed embryo transfer (FET) (n=3878)
- Natural ovulation (n=314)
- Non-complete cycle (n=171)
- In vitro maturation (IVM) or oocyte freezing (n=19)
- Preimplantation genetic screening (*PGS*) and preimplantation Genetic Diagnosis (*PGD*) (n=217); Patients after testicular sperm aspiration (TESA)-ICSI or percutaneous epididymal sperm aspiration (PESA)-ICSI (n=67).

**Fig-1:**
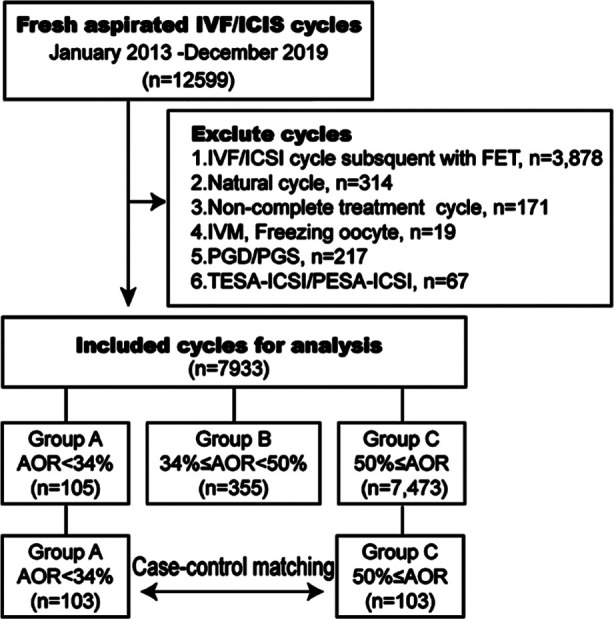
Flow chart and data processing.

The cohort included 7473 patients with AOR ≥ 50% (Group-C) and 460 patients with AOR < 50% (Group-A). The receiver operating characteristic (ROC) curve of the cutoff for AOR was used for subsequent case-control matching (Fig.1), using the SPSS statistical software to reduce the deviation of patient characteristics based on age, ovarian stimulation protocol (such as GnRH agonist protocol, GnRH antagonist protocol, etc.), and treatment (IVF or ICSI). Patients in Group-A were matched 1:1 to the patients in Group-C, 103 patients in each group (Fig.1). Age, duration of infertility, body mass index (BMI), and baseline concentrations of follicle-stimulating hormone (FSH), luteinizing hormone (LH), estradiol (E_2_), prolactin (PRL), and testosterone (T) were compared.

### Clinical stimulation protocol:

All patients received COS according to well-established stimulation protocols. The protocols were personalized based on age, infertility reason, antral follicle count (AFC), and previous cycles. Briefly, all included patients received recombinant and/or urinary gonadotropins (Gn) and underwent ultrasound and blood tests. Human chorionic gonadotrophin (hCG) 6,000-10,000 IU (trigger) was administered when 2-3 follicles reached a size of 17 mm or higher, thus indicating that the oocytes were mature.

### Oocyte retrieval and embryo transfer:

Oocyte retrieval was performed 34-36 hours after the trigger using transvaginal ultrasonography-guided aspiration. The fertilization procedure of conventional IVF or ICSI was dependent on the quality of the sperm. After three days of in vitro culture, if useable embryos were formed, the embryos were selected for transplantation according to the patient’s condition. In cases with a sufficient number of good-quality embryos on day three, blastocyst transfer was performed on day five, with the informed consent of the patient to be transplanted with fresh embryos or fresh blastocysts. Apart from the transferred embryos, the remaining embryos were vitrified on day three or during the blastocyst stage (days 5-6). Cleavage-stage embryo and blastocyst scoring was done based on the Istanbul consensus.^14^ “container-title”: “Human Reproduction (Oxford, England In all patients, vaginal progesterone was supplemented during the luteal phase. Clinical pregnancy (CP) was defined as the presence of a gestational sac on ultrasound at six gestational weeks.

### Statistical analysis:

The quantitative variables were described as the mean ± SD. For qualitative variables, the number (N) and percentage (%) for each category were displayed. Group- comparisons were performed using Student’s parametric. Proportion data were analyzed using the Chi-squared test. Multivariate logistic regression was used to assess the contributing strength of parameters that may be related to clinical pregnancy outcomes. Odds ratios (ORs) with 95% confidence intervals (CIs) were computed from the results of the logistic regression analysis. Receiver operating characteristic (ROC) curves were employed to determine the optimal sensitivity and specificity thresholds and calculate the area under the curve (AUC). Youden’s index^15^ = (sensitivity(c) + specificity(c)-1) was determined and then used to calculate the associated criterion (cutoff point). All reported p values are two-tailed. P < 0.05 indicated significance, and analyses were conducted using SPSS statistical software (v. 22.0).

## RESULTS

### Univariate analysis to identify predictors related to CP:

During the study period, a total of 12599 IVF/ICSI cycles were analyzed, and 7933 cycles (460 with an AOR<50% and 7473 with an AOR ≥ 50%) that met the inclusion and exclusion criteria were selected. The demographic and baseline characteristics of patients with an AOR <50% in relation to clinical pregnancy (CP) are shown in Table-I. Treatment characteristics, ovarian response, and oocyte parameters in different CP outcome groups are shown in Table-II. Data from the univariate analyses were used to examine the potential of the variables to predict an altered CP result. Several clinical parameters were significantly related to CP, including age, AFC, basal FSH, basal E_2_, the initial dose of Gn, duration of Gn, LH, E2, and endometrial thickness on the HCG Day. However, no significant differences were found in the analysis of BMI, the duration of infertility, basal LH, basal PRL, or basal T levels (all p>0.05).

**Table-I:** Demographic and baseline characteristics of patients with AOR <50%.

Characteristics	Clinical pregnancy (CP)		t/x^2^	P

	Yes (n=106)	No (n=354)		
No. of cycles	106	354		
Age, years	31.34±3.97	33.68±5.13	4.33	<0.001
Duration of infertility, years	4.71±2.93	4.73±3.43	0.07	0.942
BMI, kg/m^2^	21.11±2.82	21.72±3.12	1.80	0.072
AFC	16.15±8.58	11.37±7.89	5.24	<0.001
Basal FSH, IU/L	6.48±1.94	7.49±2.77	3.51	0.001
Basal LH, IU/L	3.89±1.73	3.69±1.72	1.05	0.296
Basal E_2_, pg/ml	39.76±15.72	44.32±22.23	1.97	0.049
Basal PRL, ng/ml	16.07±9.24	14.97±7.32	1.27	0.206
Basal T, ng/ml	0.33±0.15	0.32±0.15	0.62	0.538

Values are mean ± SD, BMI, body mass index; AFC, antral follicle count; FSH, follicle-stimulating hormone; LH, luteinizing hormone; E_2_, estradiol; PRL, prolactin; T, testosterone.

Women who achieved CP were younger (31.34±3.97 years vs. 33.68±5.13 years, p<0.001), while the duration of infertility years was similar between the groups. The values of AFC, basal FSH, and basal E_2_ were significantly different between women with CP and without CP (16.15±8.58 vs. 11.37±7.89, p<0.001; 6.48±1.94 vs. 7.49±2.77, p<0.001; and 39.76±15.72 vs. 44.32±22.23, p=0.049, respectively). Differences in age might explain the differences in patients’ basal hormone levels and AFC. Although the total Gn dose was not significantly different, the initial Gn dose in the pregnancy group was lower, and the time of Gn administration was longer, with statistically significant differences (189.86±45.55 IU vs. 205.26±51.15 IU, p=0.006; and 12.21±2.57 days vs. 10.81±3.17 days, p<0.001). As shown in Table-II, the number of aspirated follicles and retrieved oocytes, as well as the number of usable oocytes and the AOR, were significantly lower in non-CP patients (p < 0.001).

**Table-II:** Treatment characteristics, ovarian response and oocyte parameters of patients with AOR <50%.

Parameters	Clinical pregnancy (CP)		t/x^2^	P

	Yes (n=106)	No (n=354)		
Total Gn dose, IU	2,528.89±943.93	2,451.20±1,085.70	0.67	0.506
Start of Gn dose, IU	189.86±45.55	205.26±51.15	2.79	0.006
Duration of Gn, days	12.21±2.57	10.81±3.17	4.15	<0.001
Dose of HMG, IU	1,032.59±942.62	1,082.28±1,036.78	0.39	0.694
Duration of HMG, days	7.71±4.33	7.19±4.26	0.98	0.327
LH on HCG day (IU/L)	1.11±0.88	2.62±3.68	4.16	<0.001
E_2_ on HCG day, pg/ml	3,296.60±2,146.19	1,918.52±1,781.57	6.63	<0.001
P on HCG day, ng/mL	0.72±0.40	0.73±0.59	0.16	0.872
Endometrial thickness on HCG day, mm	11.73±1.89	10.25±2.46	5.73	<0.001
No. of follicles aspirated	10.95±5.13	6.31±5.39	7.88	<0.001
No. of oocytes retrieved	5.66±3.37	3.05±3.86	6.29	<0.001
Total available oocytes	4.25±2.20	1.84±2.23	9.78	<0.001
AOR	451/1,161(38.85)	652/2,232(29.21)	32.31	<0.001
Available oocytes/oocytes retrieved	82.40±22.19	73.92±34.94	2.31	0.021

Results are expressed as the mean ± SD or n (%), Gn, gonadotrophin; HMG, human menopausal gonadotropin; HCG, human chorionic gonadotropin.

### Multivariate logistic regression analysis of factors related to CP:

**Fig.2:**
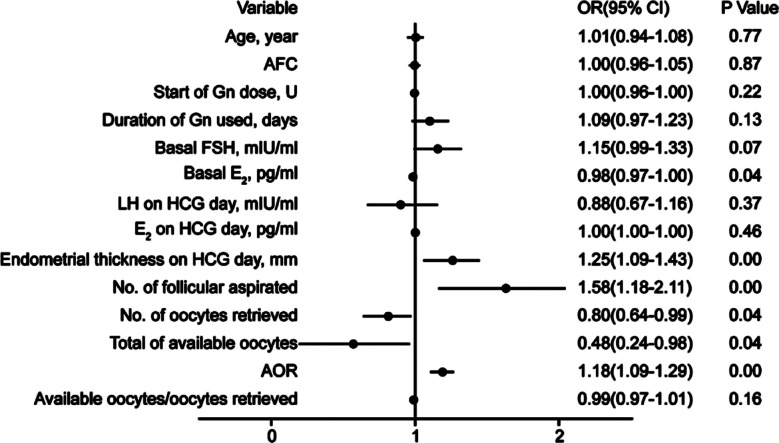
Multivariate logistic regression analysis of factors related to pregnancy elevation.

We next performed a multivariate logistic regression and ROC curves analyses of the identified predictive factors of CP. Fig.2 summarizes the results of the logistic regression model for factors that may have influenced CP. Endometrial thickness on HCG day (OR = 1.25, 95% CI: 1.09–1.43, p<0.01), the number of follicles aspirated (OR = 1.58, 95% CI: 1.18–2.11, p<0.01), and the AOR (OR = 1.18, 95% CI: 1.09–1.29, p<0.01) positively correlated with the rate of CP. On the other hand, the analysis showed an adverse effect of basal E_2_ (OR = 0.98, 95% CI: 0.97–1.00, p=0.04), the number of oocytes retrieved (OR = 0.80, 95% CI: 0.64–0.99, p=0.04), and available oocytes (OR =0.48, 95% CI: 0.24–0.98, p=0.04) on the CP outcomes. No significant effects were observed in regard to age, AFC, start of Gn dose, duration of Gn, or hormone treatment, except for basal E_2_ (all p>0.05).

**Fig.3:**
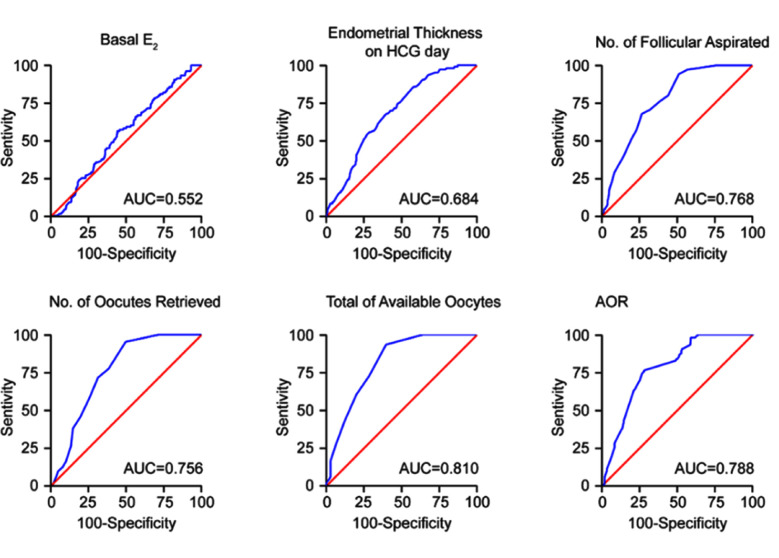
ROC (receiver operating characteristic curve) for multivariate analysis of factors related to pregnancy.

The ROC curve was used to assess the performance of the AOR for predicting clinical pregnancy compared to other parameters and showed an AUC of 0.788 for CP in patients with AOR<50%. Data on the basal E_2_, endometrial thickness, number of aspirated follicles, number of oocytes retrieved, and available oocytes are summarized in Fig.3. Except basal E_2_, most curves supported a threshold for CP, with an AUC significantly greater than 0.5. Although the total available oocytes number was associated with the largest AUC, this only considered the number of available oocytes and ignored the denominator. Therefore, the AOR was selected as a go-to step for further evaluation. The AOR cutoff value for CP prediction was 34.17%, with a sensitivity of 76.4% and a specificity of 27.1%, according to Youden’s index (YI=0.493).^10^

### Case-control matching:

A comparison of baseline characteristics between Group-A (n = 105) and Group- C (n =7,473) was then performed, as shown in Table-III. Prior to case-control matching, patient characteristics, such as duration of infertility, AFC, and basal FSH, varied significantly between the two Groups (Table III). Women in Group-A had a longer period of infertility than those in Group-C (5.19 vs 4.20, p=0.001). Mean AFC, which is the most accurate indicator for predicting ovarian response, was higher in Group-A than in Group-C (15.57 vs. 13.77, p =0.011). Basal FSH levels were significantly different between the study cohorts (6.40 vs. 6.91 mIU/ml, p =0.013). After case-control matching, the differences in baseline characteristics were analyzed for 103 matched patients (Table-III). There were no significant differences in the characteristics between the two matched Group-s, suggesting that the matched cohorts had highly similar characteristics.

**Table-III:** Demographic and baseline characteristics of patients with a low AOR (case Group-) and control Group- before and after case-control matching.

Characteristics	Before case-control matching (n=7.578)			After case-cintrol matching (n=206)		

	Group-A (n=105)	Group- C (n=7473)	p	Group-A (n=103)	Group- C (n=103)	p
Age, years	31.72±4.53	32.18±4.62	0.32	31.80±4.54	31.72±4.39	0.901
>35	31	2,234	0.934	31	30	0.879
<35	74	5,239		72	73	
Duration of infertility, years	5.19±3.37	4.20±3.03	0.001	5.19±3.39	4.42±3.05	0.085
Times of treatment	1.96±1.40	2.09±1.77	0.470	1.90±1.35	1.81±1.34	0.605
Ovarian stimulation protocol			0.839			1.000
GnRH agonist	82	5,665		82	82	
GnRH antagonist	19	1,457		19	19	
Others	4	351		2	2	
Treatment			<0.001			1.000
IVF	55	5,755		53	53	
ICSI	50	1,718		50	50	
BMI, kg/m^2^	21.43±3.03	21.54±2.90	0.712	21.45±3.06	21.48±2.97	0.858
AMH	3.58±2.72	3.29±2.69	0.627	3.33±2.56	2.87±1.82	0.270
AFC	15.57±9.28	13.77±6.89	0.011	15.47±9.25	15.58±7.10	0.924
<7	15	1,270	0.463	15	12	0.536
>7	90	6,203		88	91	
Basal FSH, IU/L	6.40±1.88	6.91±2.10	0.013	6.35±1.86	6.60±1.83	0.328
Basal LH, IU/L	3.89±2.06	3.95±1.82	0.750	3.90±2.07	4.21±1.83	0.259
Basal E2, pg/ml	46.82±21.39	43.53±19.26	0.086	46.63±21.41	41.04±19.01	0.051
Basal PRL, ng/ml	15.90±7.30	15.37±7.75	0.487	15.64±6.84	14.70±7.57	0.349
Basal T, ng/ml	0.32±0.16	0.32±0.14	0.865	0.32±0.16	0.33±0.14	0.827

Values are mean ± SD, BMI body mass index; AFC, antral follicle count; FSH, follicle-stimulating hormone; LH, luteinizing BMI BMI, body mass index; AFC, antral follicle count; FSH, follicle-stimulating hormone; LH, luteinizing hormone; E2, estradiol; PRL, prolactin; T, testosterone.

A comparison of treatment characteristics, ovarian response, and oocyte parameters is summarized in Table-IV. No differences were observed in terms of the total dose of Gn used between the two matched cohorts (2,562.74±1,004.96 IU and 2,500.31±883.72 IU). Likewise, there were no significant differences in hormone levels on HCG days in Group-A compared to Group- C (P > 0.05). However, the dose and duration of HMG used showed a statistically significant difference. Women with lower AOR received a higher dose of HMG (1023.55±984.48 IU vs. 782.29±784.54 IU, p=0.003) and a longer duration of HMG treatment (7.84±4.71 days vs. 6.38±3.96 days, p=0.024) compared to women with higher AOR. The differences in endometrial thickness and oocyte parameters are shown in Table-IV.

**Table-IV:** Treatment characteristic, ovarian response, and oocyte parameters in case-control matching Group-.

	Before case-control matching (n=7.578)			After case-control matching (n=206)		

Parameters	Group-A(n=105)	Group- C (n=7473)	p	Group-A (n=103)	Group- C (n=103)	p
Start of Gn dose, IU	194.17±48.45	199.93±50.12	0.242	194.30±48.63	198.67±49.07	0.522
Total Gn dose, IU	2.562.74±1.004.96	2.500.31±883.79	0.473	2.591.38±991.41	2.440.17±811.72	0.232
Duration of Gn, days	11.78±2.53	11.47±2.34	0.176	11.88±2.40	11.57±2.24	0.338
≥14 days	28	1.314	0.015	28	19	0.135
<14 days	77	6.159		75	84	
HMG, IU	1.023.55±984.48	782.29±784.54	0.003	1.022.18±993.22	665.53±672.89	0.004
Duration of HMG, days	7.84±4.71	6.38±3.96	0.001	7.88±4.73	5.79±3.59	0.002
LH on HCG day (IU/L)	1.25±1.02	1.56±2.51	0.212	1.21±0.90	1.22±1.02	0.913
E2 on HCG day, pg/ml	3.067.55±1.899.44	2.827.93±1.825.55	0.184	3.113.16±1.887.40	2.994.01±2.219.70	0.680
P on HCG day, ng/mL	0.86±0.51	0.79±0.44	0.106	0.86±0.51	0.82±0.44	0.540
Endometrial thickness on HCG day, mm	11.65±2.05	11.16±1.96	0.011	11.64±2.06	11.53±2.07	0.708
No. of follicles aspirated	9.86±4.79	9.91±5.10	0.911	9.87±4.82	10.55±5.72	0.357
No. of oocytes retrieved	4.41±3.71	8.85±4.77	<0.001	4.40±3.66	9.10±4.95	<0.001
AOR	214/1.035	64.288/74.081	<0.001	212/1.017(20.85)	869/1.087(79.94)	<0.001
Fertilization rate	170/463(36.72)	50.258/66.151(75.97)	<0.001	168/449(37.42)	669/937(71.40)	<0.001
Top-quality embryo	78/170(45.88)	26.851/50.258(53.53)	0.049	78/168(46.43)	365/669(54.56)	0.059
No. of ET			<0.001			<0.001
0	44	526				
1	26	1.194		26	15	
2	35	5.753		35	81	
Clinical pregnancy	17/61(27.87)	3.036/6.947(43.70)	<0.001	17/61(27.87)	41/96(42.71)	<0.001

Results expressed as mean±SD or n (%), Gn, gonadotrophin. HMG, human menopausal gonadotropin; HCG, human chorionic gonadotropin; ET, embryo transfer.

After case-control matching, differences in the dose and duration of HMG remained statistically significant (1,022.18 ± 993.22 IU vs. 665.53 ± 672.89 IU, p=0.004 and 7.88 ± 4.73 days vs. 5.79 ± 3.59 days, p=0.014, respectively). Women in Group-A also tended to have a lower number of oocytes retrieved (4.40 ± 3.66 vs. 9.10 ± 4.95, p<0.001) and a lower fertilization rate (37.42% vs. 71.40%, p<0.001), even though there was no difference between the groups regarding top-quality embryos (46.43% vs. 54.56%, p=0.059). The CP rates in Group-A were significantly lower than in Group-C (27.87% vs. 42.71%, p<0.001). The results of this analysis ultimately showed that high doses of HMG may cause lower clinical pregnancy rates (16.50% vs. 39.81%, p<0.001).

## DISCUSSION

A new concept of AOR that was introduced in this study was an important indicator of oocyte function and correlated with the CP outcome in fresh cycles. Our results indicated that AOR objectively predicts HMG as a clinically related factor that affects the number and maturation of oocytes for insemination.

In ART, the assessment of ovarian reserve and ovarian response to controlled ovarian stimulation can identify patients who may be less responsive or overreacting to exogenous gonadotropins and help individualize treatment to achieve good response and minimize the risk.^16^-^18^ At present, there are many factors that are used to predict ovarian reserve, including age and basal FSH, LH, E2, inhibin B, and AMH levels.^16^,^18^-^22^ Other measures of ovarian reserve include ultrasound measurements, such as pre-treatment ovarian volume^23^ and AFC, which is the sum of small antral follicles in both ovaries.^18^,^21^,^24^

Poor ovarian response (POR) is a pathological state in which the ovary responds poorly to gonadotropins. According to the European Society of Human Reproduction and Embryology (ESHRE) consensus, at least two of the following three features must be present to define POR: (i) advanced female age; (ii) prior POR; (iii) abnormal ovarian reserve test (ORT), or in the absence of the above criteria, two previous POR following maximal stimulation.^25^

Suboptimal ovarian response (SOR) means that in the initial period of fixed-dose FSH treatment, follicular recruitment and hormone levels are normal, and the same dose of FSH is continued from the 7th to the 10th day of the cycle. However, there is no significant increase in serum E_2_ levels or follicles.^26^ According to the diagnostic criteria, after case-control matching in Group-A, the patients were neither POR nor SOR and trended toward a large mean AFC, elevated AMH and low FSH, although the levels were not significant compared with Group-C (all p>0.05). This poses a challenge for the treating clinicians who would need to somehow identify this part of the expected normal ovarian response in infertile patients with a young age, normal hormone levels, and AMH and AFC in the normal range with poor clinical pregnancy outcomes. In this group of patients, AOR may be particularly useful as it indirectly reflects the maturation of eggs and also reminds clinicians that they should not only focus on the routine test indexes but also take appropriate measures for patients with low egg utilization (i.e., oocyte immaturity) during ovulation stimulation.

Our study describes AOR as a new tool and evaluates its ability to explore clinically related factors that affect clinical pregnancy in women undergoing IVF/ICSI. Our results showed that AOR did not completely depend on conventional ovarian reserve capacity indicators such as AMH, AFC, and basal hormones. Patients with a high AOR had better responses to gonadotropins, a higher percentage of mature oocytes for insemination, and higher rates of CP. These results demonstrate that the AOR could be an effective predictor for evaluating the ovarian response to gonadotropins.

The observed correlation between lower AOR and pregnancy rates in Group-A requires explanation. The only differences between the clinical factors identified in this study were the dose and duration of HMG. We may speculate that the observed effect may be due to the dosage of LH in HMG. The ratio of FSH to LH in the HMG component used in our center was 1:1. Therefore, a higher HMG dose contained a higher dose of luteinizing hormone. Although FSH is generally considered to be the key driving force for ovarian follicle growth and maturation, the role of LH in these processes is still controversial.^27^

As stated in the classic “two-cells-two-gonadotrophin” theory, LH is required to provide an androgen precursor for granulosa cells for estradiol biosynthesis. FSH alone can induce follicular growth, but without LH, estradiol levels remain very low, and pregnancy will not occur.^28^,^29^ Previous studies^27^,^30^ have reported that supplementing LH activity (HMG or rLH) is more effective than increasing the dose of rFSH in terms of ovarian outcomes in patients with a low response to rFSH. In some patients with a poor prognosis, the administration of rFSH combined with rLH during COS may help to increase the pregnancy rate.

Lisi et al. reported that the supplementation of LH in FSH stimulation can improve the rate of high-quality embryos in women receiving long-term agonist therapy. The concept of an LH ‘therapeutic window’ has been used in assisted reproductive technology and ovulation induction for successful conception.^31^ In this study, we found that the dose and duration of HMG were associated with the AOR (p<0.05, respectively), and we may speculate that the dose and days of the “therapeutic window” of LH contributed to the AOR. This hypothesis is supported by previous studies that show that high levels of LH may reduce the number of oocytes retrieved, the number of fertilized oocytes, and the number of available embryos, which may potentially affect the cumulative pregnancy rate.^32^-^35^

Our results imply that low LH concentrations, therefore, may be needed for oocyte quality and the subsequent embryonic development, and reducing the proportion of LH in HMG, such as the 2:1 formulation of recombinant human FSH and recombinant human LH,^29^ may influence the number of oocytes retrieved and ultimately be beneficial for women receiving ovarian stimulation for ART. Moreover, for patients < 35 years old with normal gonadotropin levels, FSH alone can potentially be used for ovarian stimulation.

### Limitations:

This is a retrospective single-center study with a limited sample size. This introduces a potential risk of bias, despite the fact that the risk differences among different AOR groups were consistent after case-control matching. Another confounding variable that may place restrictions on the generalized application of the results is that this analysis paid close attention to the influence of HMG, as the impact of different gonadotrophin preparations for ovarian stimulation on the therapeutic effect has been widely debated.

This study also did not look at the combination of Gn to deeply explore the impact of different Gn types and different HMG sources on the research results due to the limitation of sample size. Compared with HMG alone, the combined use of rFSH and rLH tends to increase the number of available embryos and the pregnancy rate. Additional factors not mentioned in this study, such as maturation defects of oocytes,^36^ iatrogenic injury^37^ (i.e., ovarian surgery, history of radiotherapy and chemotherapy), low HCG response,^38^ and improper trigger timing^39^ may also affect the AOR. Further multi-center, prospective studies with large sample sizes are needed to eliminate the potential confounding effects and draw a more representative conclusion for the general population.

## CONCLUSION

The AOR was an effective indicator of COS efficiency before insemination in fresh cycles. HMG is an independent clinical factor of the AOR that influences the clinical pregnancy rate in IVF/ICSI cycles. In couples undergoing IVF/ICSI with the expected normal ovarian response, higher AOR is associated with a greater number of retrieved oocytes, higher fertilization rate, and higher clinical pregnancy rate.

### Authors’ Contributions:

**BHZ:** Conceived and designed the study, responsible for the accuracy or integrity of the work.

**SQZ**, **RSL**, **XQZ** and **HLX:** Contributed to the clinical data collection.

**PYL** and **RSL:** Contributed to the statistical analysis and interpretation of the data.

**PYL:** Drafted the manuscript and **SQZ** finalized it.

All the authors contributed to the critical revision and final approval of the manuscript.
